# Macular Structure–Function Relationships of All Retinal Layers in Primary Open-Angle Glaucoma Assessed by Microperimetry and 8 × 8 Posterior Pole Analysis of OCT

**DOI:** 10.3390/jcm10215009

**Published:** 2021-10-28

**Authors:** Jose Javier Garcia-Medina, Maurilia Rotolo, Elena Rubio-Velazquez, Maria Dolores Pinazo-Duran, Monica del-Rio-Vellosillo

**Affiliations:** 1 Department of Ophthalmology, General University Hospital Morales Meseguer, 30007 Murcia, Spain; erubiovelazquez@gmail.com; 2 Department of Ophthalmology, General University Hospital Reina Sofia, 30003 Murcia, Spain; rotolomaurilia@gmail.com; 3 Department of Ophthalmology and Optometry, University of Murcia, 30120 Murcia, Spain; 4 Ophthalmic Research Unit Santiago Grisolia, 46017 Valencia, Spain; pinazoduran@yahoo.es; 5 Red Temática de Investigación Cooperativa en Patología Ocular (OFTARED), Instituto de Salud Carlos III, 28029 Madrid, Spain; 6 Department of Ophthalmology, University of Valencia, 46010 Valencia, Spain; 7 Department of Anesthesiology, University Hospital Virgen de la Arrixaca, 30120 Murcia, Spain; monicadelriov@hotmail.com

**Keywords:** microperimetry, OCT, glaucoma, macula, structure, function, correlation, retina, inner, outer

## Abstract

Purpose**:** The aim of this study is too correlate the sensitivity and thickness values of intraretinal layers at macula in healthy eyes and primary open-angle glaucoma (POAG) eyes. Methods**:** The thickness of different intraretinal segmentations was estimated by means of optical coherence tomography (OCT) Spectralis (Heidelberg, Engineering, Inc., Heidelberg, Germany) with the posterior pole analysis program 8 × 8 in 91 eyes from 91 patients (60 with glaucoma and 31 healthy patients). Macular sensitivity was also measured with an MP-1 microperimeter (Nidek Instruments, Inc Padova, Italy) with a customized, 36-stimulus pattern adjusted to an anatomical correspondence with the OCT grid. Correlations were calculated by using Spearman’s rho and the results were represented in color maps. Results**:** Significant structure–function correlations were much more frequent in the glaucoma group than in control group. In general terms, associations were positive for inner retinal layers but negative correlations were also found for the inner nuclear layer and outer retinal layer in glaucoma. Conclusions: In general terms, significant structure–function correlations for different intraretinal layers are higher and wider in POAG eyes than in healthy eyes. Inner and outer retinal layers behave differently in terms of the structure–function relationship in POAG as assessed by microperimetry and OCT.

## 1. Introduction

Glaucoma is a chronic and progressive neuropathy of the optic nerve characterized by retinal ganglion cell apoptosis [1]. This event is not only associated with optic nerve cupping due to retinal nerve fiber layer loss (dendrites of the retinal ganglion cells) but also with changes at different intraretinal layers of the macula as shown in our previous studies [2,3].

All these structural modifications are usually accompanied by functional losses that can be first observed as localized visual field (VF) defects or scotomas that can affect the macular region [4].

The structure–function relationship has been widely studied. However, most of the investigations have been functionally performed with standard automated perimetry (SAP) [5].

Microperimetry (MP) is a technology with theoretical benefits in relation to SAP such as automated eye tracking and more precise location of the stimulus on the retina [6]. However, few works have studied the macular structure–function relations in glaucoma using MP [7,8,9,10]. Plus, all of them have only structurally analyzed some of the inner retinal segmentations of the macula obtained by optical coherence tomography (OCT). Although glaucoma is primarily a disease of ganglion cells, in the light perception and early integration of visual stimuli all retinal layers participate, not only in inner retinal layers. Considering the structural changes detected by our group in glaucoma [2,3], we hypothesize that there could exist associations between thickness of all retinal layers with its anatomically corresponding sensitivity in glaucoma and, specifically, with such a precise technology as MP. To the best of our knowledge, we have not found any study in this sense. Thus, this is the purpose of the present work.

## 2. Materials and Methods

This is a prospective, observational, cross-sectional study. All the eyes included in the study were recruited from consecutive patients seen at the Glaucoma Clinic of General University Hospital Reina Sofia, Murcia, Spain. The investigation was approved by the institutional review board of the mentioned hospital. All participants signed an informed consent and the study was conducted in accordance with the tenets of the Declaration of Helsinki.

The inclusion criteria of glaucomatous group were as follow:

1. Caucasian race and age between 40 and 90 years;
2. Diagnosis of primary open-angle glaucoma (POAG) with threat to fixation according to our clinical records;
3. Refractive error of 3 dioptres or less of spherical equivalent and;
4. Best corrected visual acuity of 0.5 or better in the Snellen scale.

POAG diagnosis required:

1. Intraocular pressure > 21 mmHg in at least three different days;
2. Glaucomatous optic disc changes and/or characteristic glaucomatous SAP abnormalities, as judged by a glaucoma specialist (J.J.G.M.), and;
3. Open anterior chamber angle in gonioscopy.

Threat to fixation was defined as a depression of one or more of the four paracentral points with a *p* < 1% in the two previous reliable 30-2 SITA Fast VF tests [11]. SAPs were considered reliable when loss of fixation, false-positive and/or false-negative responses were under 20% and were obtained from clinical records in the past year.

Inclusion criteria for healthy group (controls) were the same that for glaucomatous group except for criterium number 2. Exclusion criteria for both groups were as follow:

1. Previous intraocular or refractive surgery, or laser procedure in the six months before the recruitment;
2. History of ocular trauma;
3. Use of ocular or systemic medications that could affect the VF;
4. Presence of other ophthalmic or systemic significant diseases (eyelid, corneal, lens or retinal disease, diabetes) that could influence microperimetry or OCT results.

Only one eye was selected per patient. When both eyes were eligible one of them was randomly chosen.

At the time of enrollment, an ophthalmic examination was performed that included the following tests, which were made in this order:

1. Autorefractometry;
2. Best Corrected Visual Acuity (BCVA), using decimal scale;
3. Intraocular pressure estimation using applanation tonometry.

Microperimetry and OCT examinations were performed in a subsequent visit if the patient was eligible.

Microperimetry was conducted with the MP-1 (Nidek Instruments Inc., Padova, Italia) using a customized pattern by the same examiner (M.R.) selecting a full-*threshold strategy* 4-2-1 decibels with a Goldmann III-size stimulus presented for 200 milliseconds and a 4 apostilb backgroud. The maximal sensitivity for each stimulus was 20 decibels.

This pattern consisted of 37 stimuli centered at the fovea and with the following features:

1. The nearest stimuli from vertical and horizontal main axes were located at 1.5 degrees from these axes;
2. The stimuli were separated 3 degrees from each.

**Figure 1:**
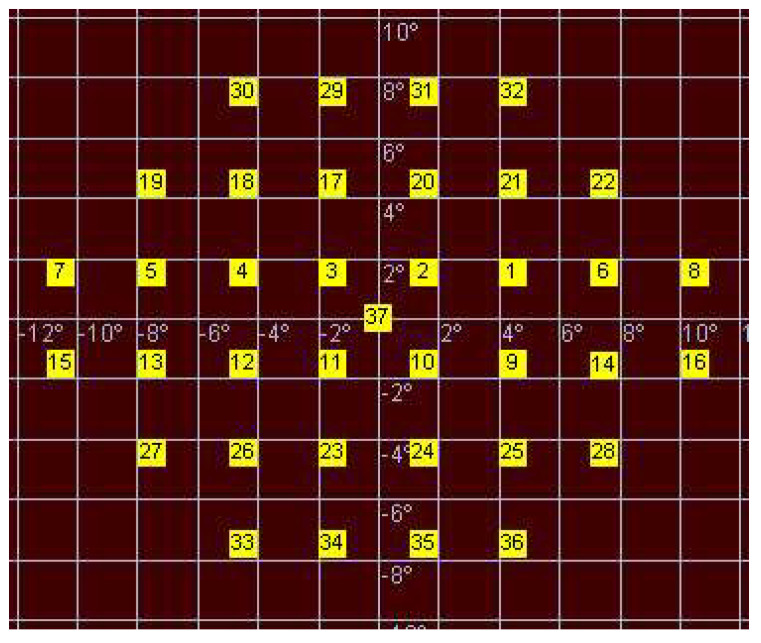
Customized pattern of microperimetry used in this study.

The central stimulus (number 37), located in the intersection of the main vertical and horizontal axes, was not considered for the calculations (Figure 1).

**Figure 2:**
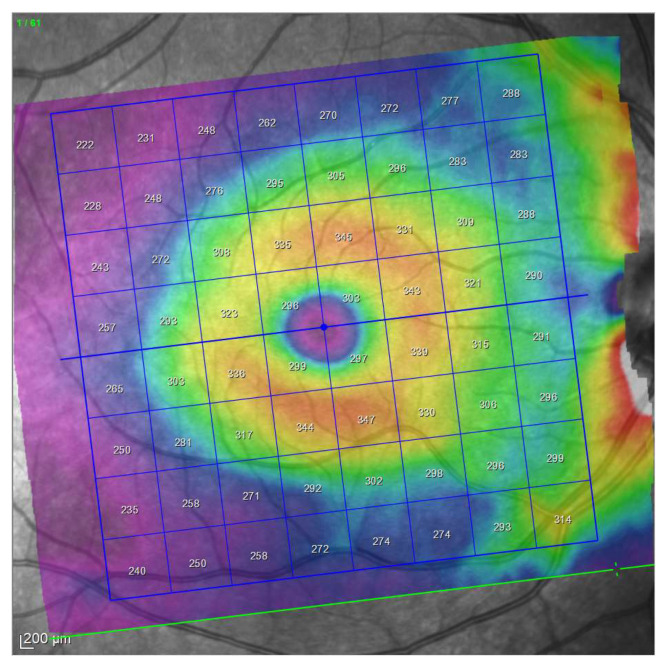
Posterior pole program analysis. Note the inclination of the grid automatically generated by the device.

Then OCT examinations were performed using an 8 × 8 posterior pole algorithm with the device Spectralis (Heidelberg Engineering, Heidelberg, Germany; 6.0 software version). This algorithm estimates the thickness of the considered layer of 64 superpixels centered at the fovea. Each superpixel is 3 × 3 degrees wide. Alignment of the horizontal main axis according to the fovea—disc axis is automatically displayed in this algorithm (Figure 2).

**Figure 3:**
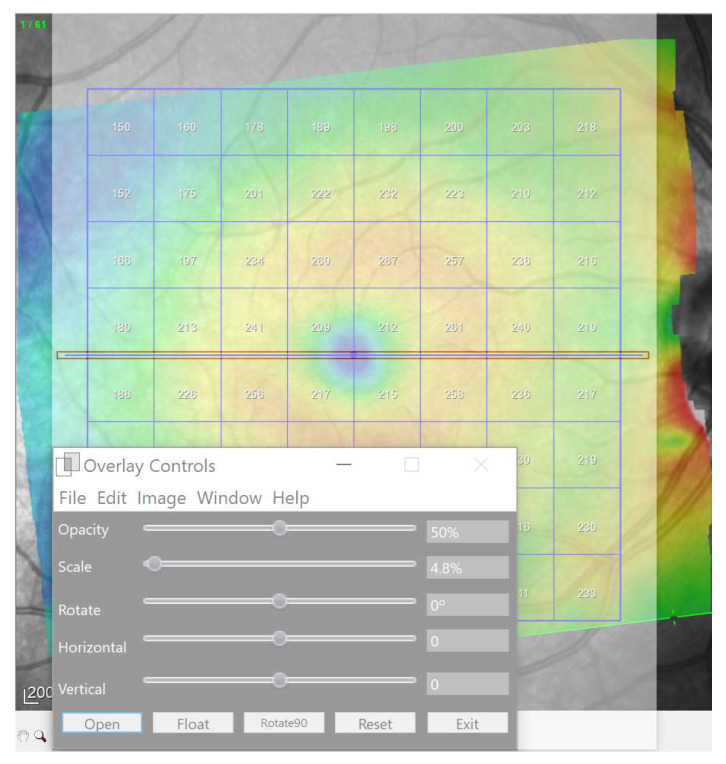
Horizontalization of the grid using a overlaid reference horizontal rectangles (red) by means of Overlay 2.1 software.

In the present study, the 8 × 8 grids were horizontalized using a reference horizontal rectangle at zero degrees overlaying on the OCT interface by means of the software Overlay 2.1 (Collin Thomas Photography Ltd. London, UK, http://www.colinthomas.com/overlay (accessed on 25 October 2021)). (Figure 3).

**Figure 4:**
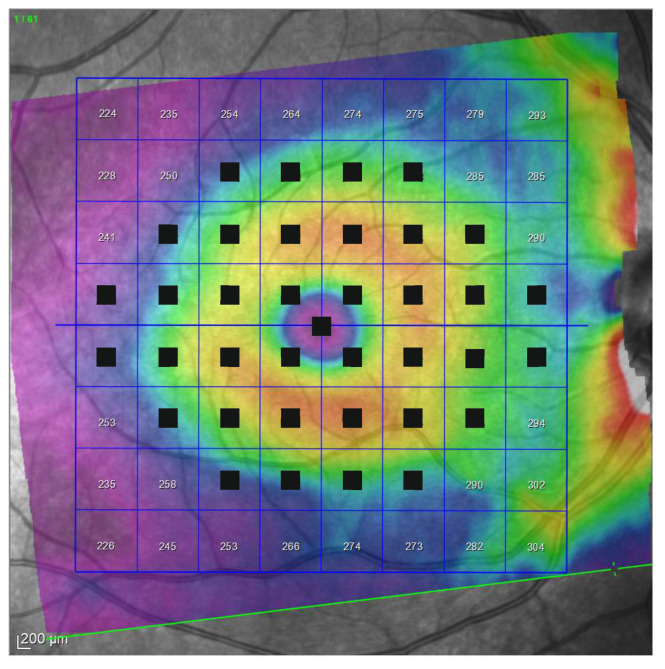
Horizontal OCT grid and microperimetry pattern.

Thus, using this customized pattern in microperimetry, we located each stimulus at the center of each superpixel of the horizontalized grid in the OCT 8 × 8 posterior pole grid to achieve the anatomical correspondence (Figure 4).

**Figure 5:**
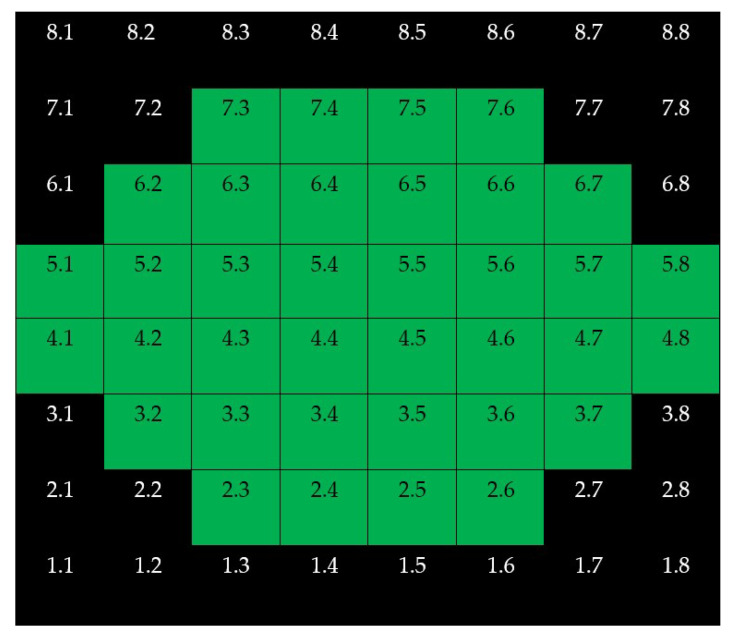
Denomination of superpixels in 8 × 8 Posterior Pole Algorithm (right eye). Only the thickness of the superpixels represented in green (and its corresponding microperimetric sensitivities) were considered in this study. All eyes were represented as if they were all right eyes.

Only superpixels with a projected stimulus at the center were considered in this study. All maps were constructed as if all eyes were right ones (Figure 5).

Only reliable OCT examinations (with signal strength ≥ 20) were selected. All scans were checked by the same experienced operator (J.J.G.M.). If segmentation errors were detected, the examinations were considered unreliable and discarded.

**Figure 6:**
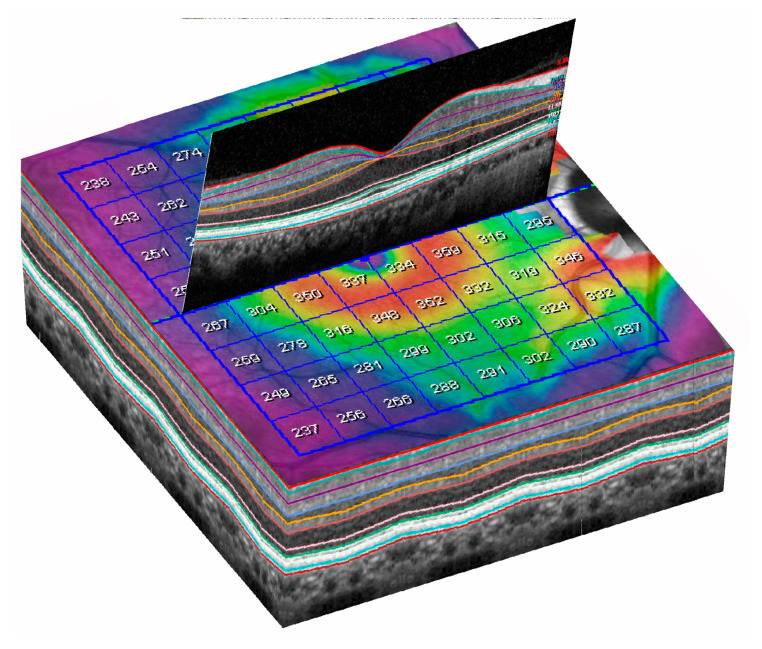
Three-dimensional (3D) representation of automatic segmentation of all retinal layers in a 8 × 8 grid.

Spectralis OCT allows an automatic segmentation of different intraretinal layers (Figure 6) and estimation of thickness of the selected segmentation.

In this study the thickness of the following automatic segmentations were considered at the macula: full retina, retina nerve fiber layer (mRNFL)**,** ganglion cell layer(GCL), inner plexiform layer (IPL), inner nuclear layer (INL), outer plexiform layer (OPL), outer nuclear layer (ONL), outer retina, and retinal pigment epithelium (RPE). ONL and OPL thickness values were added (OPL + ONL) in order to avoid artefactual results due to Henle fibers orientation [12].

Statistical calculations were performed using SPSS software (IBM, version 22, Chicago, IL, USA). The sex and laterality of the eye were compared using Fisher’s test between groups. All the continuous variables were assessed for normality with the Kolmogorov–Smirnov test. Age, clinical variables, all microperimetric results (in decibels), and many of the thickness OCT results (in microns) did not show a normal distribution. Thus, comparisons of mean age, BCVA, spherical equivalent, IOP, and vertical cupping between both groups were assessed by means of the Mann–Whitney test. Correlations between the sensitivity and thickness for each segmentation at each megapixel were calculated by Spearman’s rank correlation coefficient. structure–function correlation maps were plotted for each considered segmentation. All were constructed as if all eyes were right eyes. The significance level was *p* < 0.05.

## 3. Results

Sixty eyes of 60 POAG patients and thirty-one eyes of 31 controls were finally selected. Demographic and clinical data are presented in Table 1.

**Figure 7:**
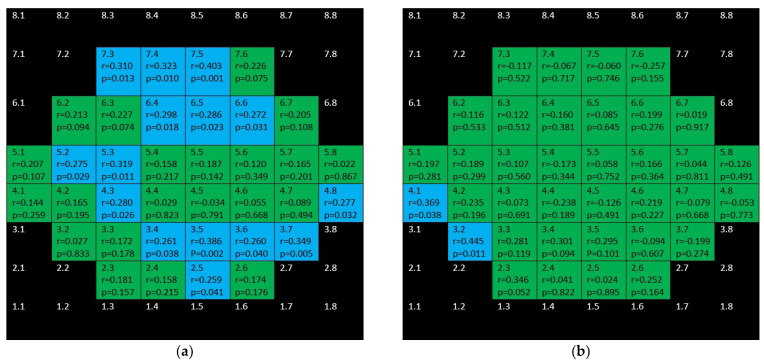
Color map of correlations for full retinal thickness in the glaucoma group (**a**) and control group (**b**). Green color indicates non-significant correlation. Blue color indicates significant positive correlation. Red color indicates significant negative correlation. Black color indicates OCT superpixels not considered in the calculations. r = Spearman’s rho, *p* = significance.

Figure 7 sums up the structure function associations between full retina thickness and microperimetric sensitivity both in the control group and the glaucoma group. In the glaucoma group positive, several moderate–weak correlations were found in the superior and inferior hemisphere but not affecting the central area. There were only two positive correlations in the control group.

**Figure 8:**
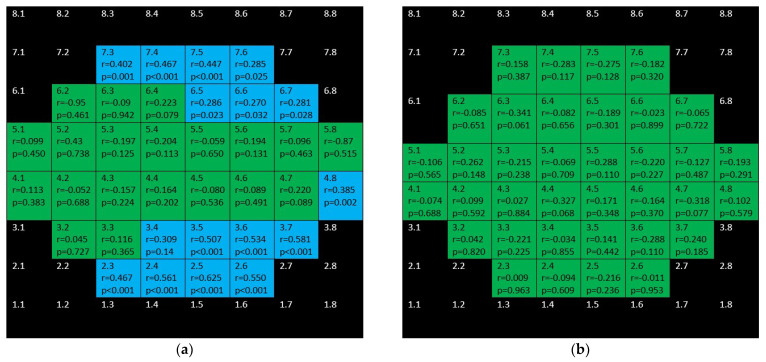
Color map of correlations for macular retinal nerve fiber layer in the glaucoma group (**a**) and control group (**b**). Green color indicates non–significant correlation. Blue color indicates significant positive correlation. Red color indicates significant negative correlation. Black color indicates OCT superpixels not considered in the calcula-tions. R = Spearman’s rho, *p* = significance.

mRNFL showed positive, weak–moderate correlations in the superior hemisphere and strong–moderate ones in the inferior hemisphere in the glaucoma group but none in the control group (Figure 8).

**Figure 9:**
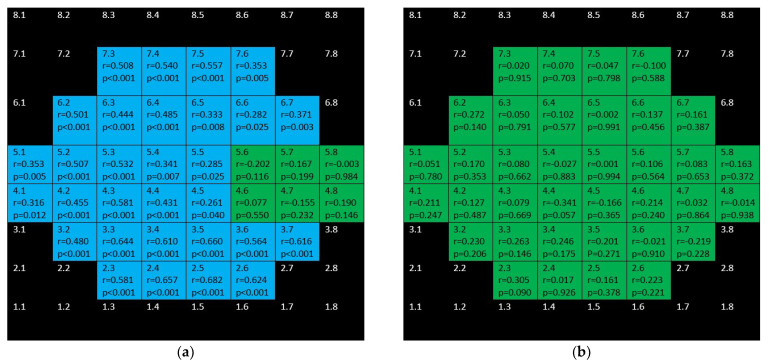
Color map of correlations for ganglion cell layer in the glaucoma group (**a**) and control group (**b**)**.** Green color indicates non-significant correlation. Blue color indicates significant positive correlation. Red color indicates significant negative correlation. Black color indicates OCT superpixels not considered in the calculations. r = Spearman’s rho, *p* = significance.

**Figure 10:**
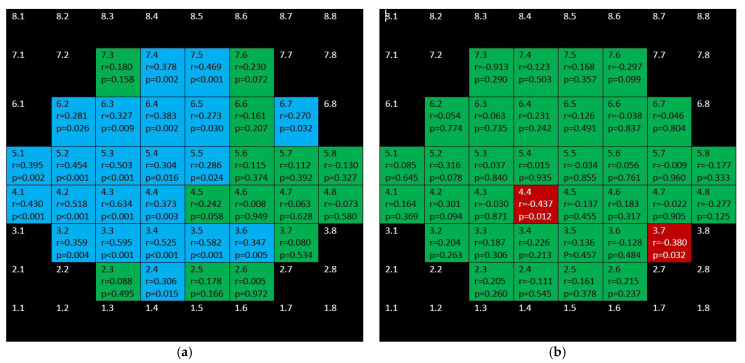
Color map of correlations for inner plexiform layer in the glaucoma group (**a**) and control group (**b**). Green color indicates non-significant correlation. Blue color indicates significant positive correlation. Red color indicates significant negative correlation. Black color indicates OCT superpixels not considered in the calculations. r = Spearman’s rho, *p* = significance.

Figure 9 and Figure 10 depict the correlations corresponding to GCL and IPL, respectively. In the glaucoma group, as it can be seen, difuse, positive, strong–moderate correlations were demonstrated in the field except for papillomacular bundle area. These correlations were stronger and more difuse for GCL than for IPL. In the control group, no significant association were found for GCL and scarce but negative ones were shown for IPL.

**Figure 11:**
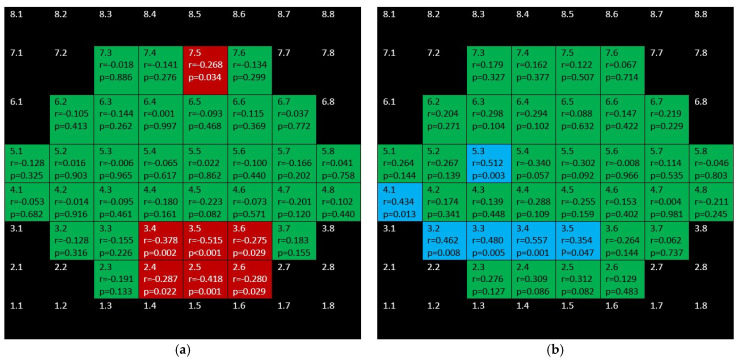
Color map of correlations for inner nuclear layer in the glaucoma group (**a**) and control group (**b**). Green color indicates non-significant correlation. Blue color indicates significant positive correlation. Red color indicates significant negative correlation. Black color indicates OCT superpixels not considered in the calculations. r = Spearman’s rho, *p* = significance.

Figure 11 shows the completely different structure–function behavior for INL in both groups. While there were positive correlations in the glaucoma group, the control group showed negative correlations.

**Figure 12:**
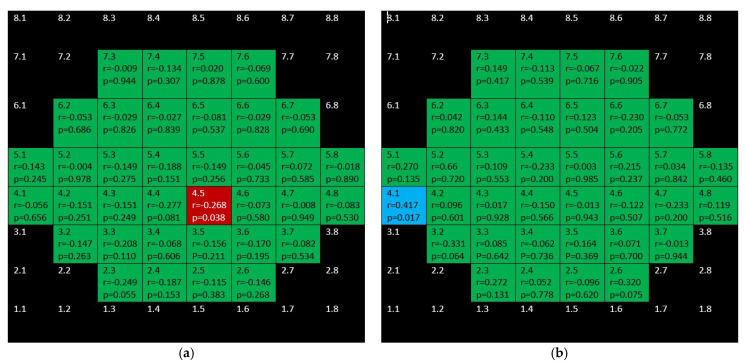
Color map of correlations for outer plexiform layer + outer nuclear layer in the glaucoma group (**a**) and control group (**b**). Green color indicates non-significant correlation. Blue color indicates significant positive correlation. Red color indicates significant negative correlation. Black color indicates OCT superpixels not considered in the calculations. r = Spearman’s rho, *p* = significance.

Figure 12 shows that barely any significant correlation was found in both groups when considering OPL + ONL.

**Figure 13:**
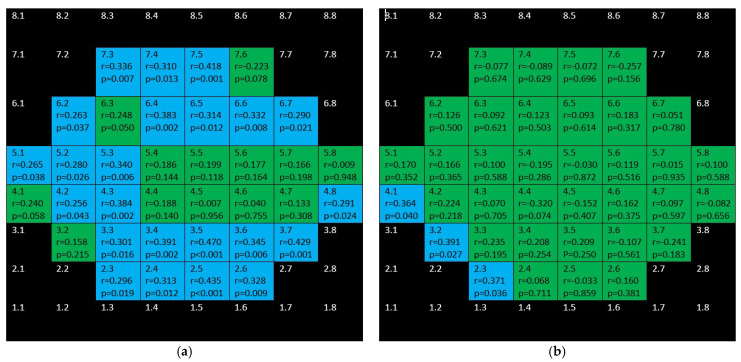
Color map of correlations for inner retinal layer in the glaucoma group (**a**) and the control group (**b**). Green color indicates non-significant correlation. Blue color indicates significant positive correlation. Red color indicates significant negative correlation. Black color indicates OCT superpixels not considered in the calculations. R = Spearman´s rho, *p* = significance.

Positive, ring-shape correlations for the glaucoma group were seen when considering inner retina (mRNFL + GCL + IPL + INL + OPL + ONL) and not affecting the to papillomacular bundle (Figure 13). Only three positive correlations were found in control the group.

**Figure 14:**
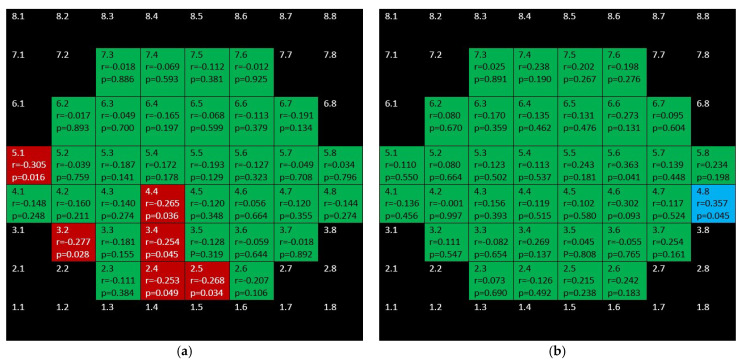
Color map of correlations for outer retina layer in the glaucoma group (**a**) and control group (**b**). Green color indicates non-significant correlation. Blue color indicates significant positive correlation. Red color indicates significant negative correlation. Black color indicates OCT superpixels not considered in the calculations. r = Spearman’s rho, *p* = significance.

Figure 14 depicts that while there were significant, negative correlations for outer retina (including outer photoreceptors segments and RPE) in the inferior hemisphere of the glaucoma group, there was only one positive correlation in the control group.

**Figure 15:**
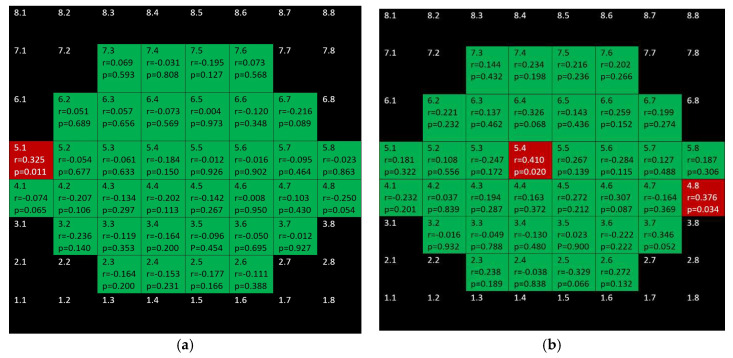
Color map of correlations for retinal pigment epithelium in the glaucoma group (**a**) and control group (**b**)**.** Green color indicates non-significant correlation. Blue color indicates significant positive correlation. Red color indicates significant negative correlation. Black color indicates OCT superpixels not considered in the calculations. r = Spearman’s rho, *p* = significance.

Finally, there are isolated correlations for RPE in both glaucoma and control group (Figure 15).

## 4. Discussion

This study dealt with the structure–function correlation of all retinal layers using OCT and MP. As mentioned in the introduction, a number of studies about the structure–function relationship have been performed by means of SAP [5]. SAP is a technique in which the projection of the stimulus on the retina can vary anatomically due to head misalignments and fixation losses. In contrast, MP is a technique that ensures that the projection of the stimulus is exactly at a specific anatomical point [6]. This is possible because MP has a real-time eye tracking system that stops the test until the eye is exactly oriented as it should be. This fact is controlled because the device continuously detects anatomical references that should be correctly positioned and only in this case does the examination go on. Plus, MP has been shown in some studies to have better ability to detect VF defects in comparison with SAP [13,14].

Only few studies have investigated macular structure–function correlation with MP in glaucoma. Some authors demonstrated that there were strong–moderate positive correlations between retinal sensitivity measured by microperimetry and ganglion cell layer + inner plexiform layer (GCLIPL) thickness in all the sectors and the sector average of the macular ellipsoid implemented in the Cirrus OCT device [7,8,9]. Zabel et.al. also found good positive correlations between the mean ganglion cell complex (mRNFL + GCL + IPL) thickness of the complete macula as determined by OCT and the average sensitivity threshold in a study aiming to correlate OCT angiography results and microperimetry results [10]. The results in this study are coincident with these mentioned studies in relation to ganglion cell-related layers showing positive, strong–moderate correlations. However, not only inner retinal layers are affected in glaucoma. Experimental studies [15,16] and human studies [2,3] have shown that outer retinal layers are also altered in glaucoma. However, to the best of our knowledge, the structure–function relationship for outer retinal layers has not been previously studied with MP.

In our study, they are remarkable the negative correlations found for outer retinal layer (photoreceptors + RPE) in the glaucoma group. This could be in relation to the changes found in these layers in our previous works [2,3] and these findings merit further investigation. Another noteworthy result in this study is the behavior of the structure–function relationship in the inner nuclear layer: while in the control group positive and moderate relationships were demonstrated, in the glaucoma group, the relationships found were also moderate but negative.

In general terms, the correlations found in this study were larger in glaucoma group than in the control group for almost all the considered retinal segmentations achieving the most intense associations for GCL and mRNFL and specially in the inferior hemisphere.

In this study we selected glaucomatous eyes with a threat to fixation in order to make sure that there was macular alteration in glaucomatous eyes. When scotomas progress towards the VF center in glaucoma, they may ultimately threaten fixation. Threat to fixation is considered an alarm sign in the management of patients with glaucoma because its progression may result in a significant decrease in vision-related quality of life [11,17,18,19]. However, threaten to fixation is not unusual in patients suffering from glaucoma. It has been described that threat to fixation is present at the diagnosis of glaucomain almost 60% of eyes [20].

This study presents several limitations to be considered. One was the relatively small size of the sample. Another one was the fact that the glaucoma group was made up of Caucasic patients suffering from POAG, so our results might not be extrapolated to other ethnic groups or to other types of glaucoma. Otherwise, each MP Goldmann III-size stimulus (circular light spot with a diameter of 43 degrees) [21] is projected at the center of a 3 × 3 degree OCT superpixel so the anatomical correspondence is not exact but it is representative of the studied area. Plus, it has been recently show that the MP-1 device has an overall bias in sensitivity values, as well as an eccentricity based measuring anomaly so our results may have been influenced by these facts [22]. Additionally, the fact that the strength of a correlation changes with the range of measurements being considered [23] could have induced, at least in part, the stronger correlations seen in the glaucoma group where both sensitivities and thicknesses change over a greater range. Finally, this is a cross-sectional study so, due to its nature, this investigation does not permit to study progressive changes of POAG.

## 5. Conclusions

In general terms, significant structure–function correlations for different intraretinal layers are higher and wider in POAG eyes than in healthy eyes and it is more marked for inner retinal layers as determined by using MP and OCT. Additionally, inner and outer retinal layers behave differently in terms of the structure–function relationship in POAG.

## Figures and Tables

**Table 1:** Demographic and clinical data of this study. Significant results are indicated in bold.

Demographic and Clinical Data	Glaucoma Group	Control Group	Significance (Test)
Eyes and laterality	*n* = 60 Right eyes = 30 Left eyes = 30	*n* = 31 Right eyes = 15 Left eyes = 16	1 (Fisher’s test)
Age (years)	73 (15)	67 (31)	0.07 (Mann–Whitney test)
Sex	29 men 32 women	9 men 22 women	0.11 (Fisher’s test)
BCVA	0.9 (0.3)	1 (1.9)	0.07 (Mann–Whitney test)
IOP (mmHg)	20 (7)	17 (4)	0.004 (Mann–Whitney test)
Spherical equivalent	0.25 (4)	1 (2)	0.04 (Mann–Whitney test)
Vertical cupping	0.8 (0.3)	0.4 (0.2)	<0.001 (Mann–Whitney test)

Continuous variables are reported with median (interquartile range).
